# Functional enhancement strategies for immunomodulation of mesenchymal stem cells and their therapeutic application

**DOI:** 10.1186/s13287-020-01920-3

**Published:** 2020-09-14

**Authors:** Byung-Chul Lee, Kyung-Sun Kang

**Affiliations:** 1 Translational Stem Cell Biology Branch, National Heart, Lung, and Blood Institute, National Institutes of Health, Bethesda, MD 20892 USA; 2 Adult Stem Cell Research Center and Research Institute for Veterinary Science, College of Veterinary Medicine, Seoul National University, 1 Gwanak-ro, Gwanak-gu, Seoul, 08826 Republic of Korea

**Keywords:** Mesenchymal stem cells, Preconditioning, Hypoxia, 3D culture, Genetic modification, Co-administration

## Abstract

Mesenchymal stem cells (MSCs) have recently been considered a promising alternative treatment for diverse immune disorders due to their unique biomedical potentials including the immunomodulatory property and ability to promote tissue regeneration. However, despite many years of pre-clinical studies in the research field, results from clinical trials using these cells have been diverse and conflicting. This discrepancy is caused by several factors such as poor engraftment, low survival rate, and donor-dependent variation of the cells. Enhancement of consistency and efficacy of MSCs remains a challenge to overcome the current obstacles to MSC-based therapy and subsequently achieve an improved therapeutic outcome. In this review, we investigated function enhancement strategies by categorizing as preconditioning, genetic manipulation, usage of supportive materials, and co-administration with currently used drugs. Preconditioning prior to MSC application makes up a large proportion of improvement strategies and preconditioning reagents include bioactive substances (cytokines, growth factors, and innate immune receptor agonists), hypoxia, and modification in culture method. With the piled results from previous studies using each method, disease- or patient-specific therapy has become more important than ever. On the other hand, genetic manipulation targeting therapeutic-associated factors or co-administration of biocompatible materials has also arisen as other therapeutic strategies. Thus, we summarized several specialized tactics by analyzing up-to-date results in the field and proposed some promising enhancement methods to improve the clinical outcomes for MSC therapy.

## Background

**Fig. 1:**
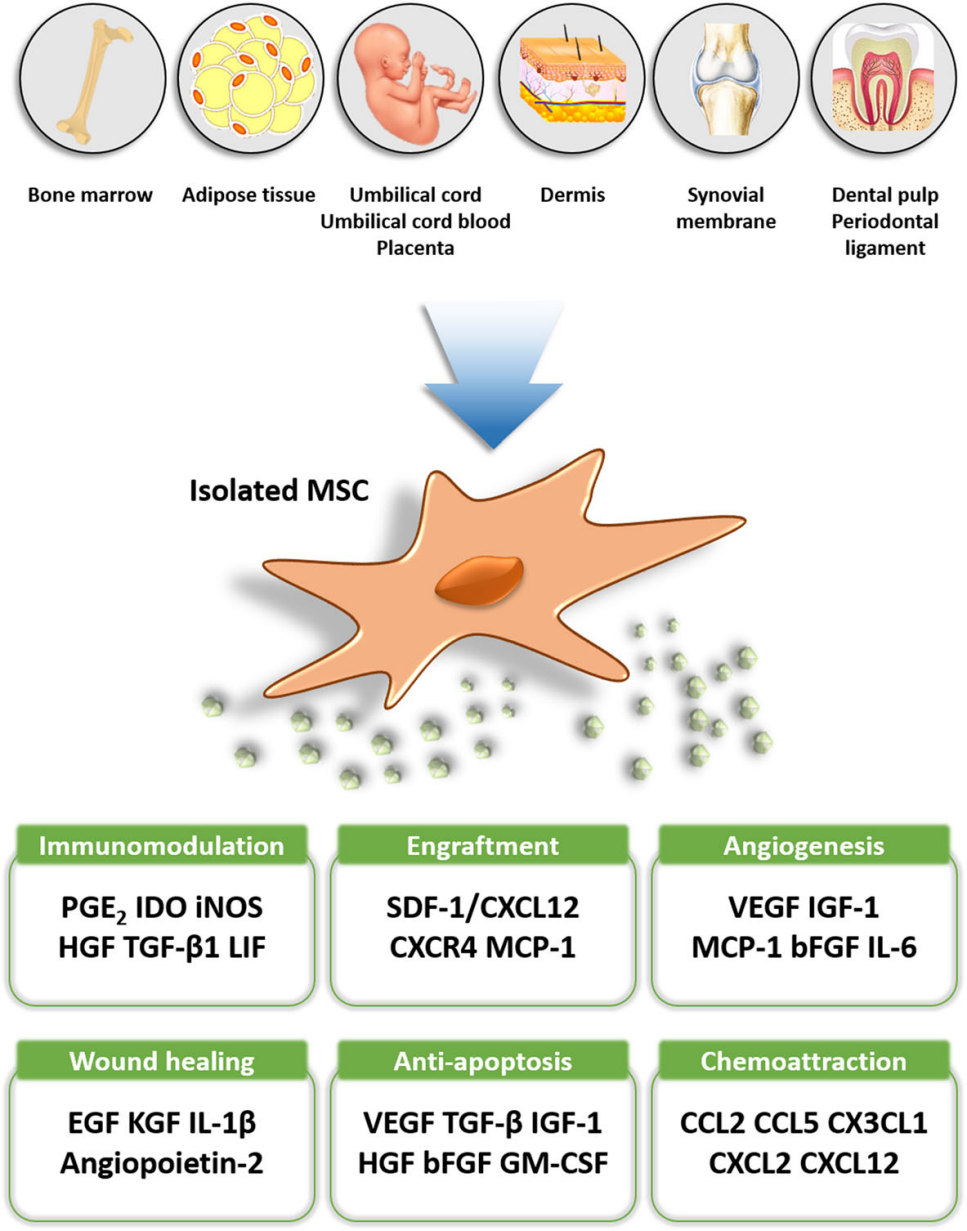
Isolation sources and therapeutic function of MSCs. A schematogram illustrating various sources for the isolation of MSCs and biological properties associated with their therapeutic effects. For decades of MSC research, alternative sources for cell isolation have been developed to avoid invasiveness mediated by bone marrow aspiration. MSCs have various and unique therapeutic potentials, which could be pleiotropic or adjustable to each disease target

Mesenchymal stem cells (MSCs) have been considered a versatile source for cell therapies due to their distinctive features including immunomodulation, angiogenetic function, wound repair, and mobilization into inflamed sites in response to the cytokines or chemokines released from lesions (Fig. 1). Given their unique therapeutic potentials, MSCs have been clinically applied to the treatment of several rare diseases such as bone and cartilage diseases [1, 2], diabetes mellitus (DM) [3], neurodegenerative diseases, and acute brain injury [4] for decades. Since the first clinical trial using bone marrow (BM)-MSCs performed in 1995 [5], numerous clinical trials have been conducted, and a total of 1081 interventional types of studies targeting a very wide range of diseases are enrolled on the public clinical database (http://www.clinicaltrials.gov).

**Fig. 2:**
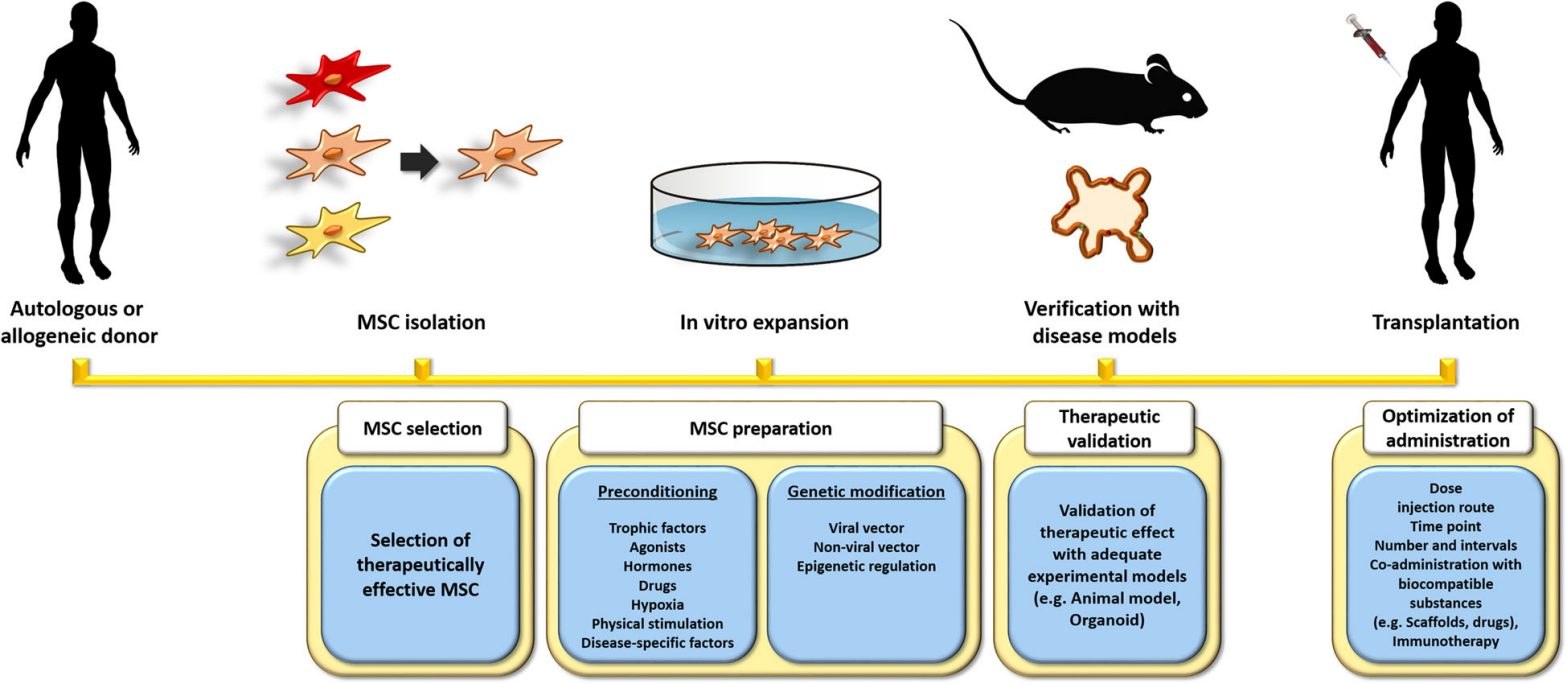
Comprehensive management of the production of hMSCs for transplantation. Isolated MSCs should be selected based on the analysis of the gene-wide profile. Selected MSCs are cultured with preconditioning factors, especially key molecules in the pathogenesis of the target disease, and during the period, the property of the selected cells has to be maintained. Also, the therapeutic function of MSCs can be enhanced by genetic modification. The therapeutic function is repeatedly validated with proper disease models. To improve the therapeutic outcomes, optimizing the condition of administration such as the adequate time point is important, and MSCs are able to be applied with biocompatible substances or advanced medical technologies

Although therapeutic potentials of MSCs have been demonstrated through preclinical researches, several consequences from clinical trials could not satisfy the patients. These discrepancies are mediated from the limitations such as poor engraftment, in vitro senescence, functional quiescence after the application, and donor-dependent variation. Since better alleviation strategies with MSC therapy are still required, various enhancement strategies have been suggested to maintain the stemness of MSCs, as well as augment the therapeutic efficacy after the infusion. To accomplish the advancement of adult stem cell-based therapy, different time points in the preparatory process were aimed for integrated management. Herein, we investigated various enhancement methods targeting each preparatory step by manipulating MSC properties (Fig. 2), such as in vivo survival, engraftment, and immunomodulatory function, for the successful therapeutic application. In the present study, we sought to conduct comprehensive analyses on enhancement strategies using various source-derived MSCs for the feasibility of MSC-based therapy.

## Main text

### Enhancement strategies for MSC-based therapy

To achieve successful therapeutic outcomes, several advanced strategies for MSC application have been proposed for decades. First of all, the selection of adequate cell candidates should be preceded. Notably, preconditioning of MSCs before application by treating cytokines or modifying the culture method is one of the readily applicable strategies, which augment immunosuppressive capacity or in vivo cell survival through paracrine effects. Next, genetic modification would be a powerful therapeutic strategy. The last one is a co-administration with bioactive materials including immunosuppressants or a cell scaffold to enhance in vivo cell survival and homing.

### MSC selection

During the preparatory procedure for transplantation, MSC selection is the first consideration we have met. At the beginning of preclinical and clinical research, it was needed to investigate whether infused MSCs could occur systemic or local immune responses. Given that MSCs were proved to avoid recipients’ immune surveillance, other factors that affect the therapeutic potential, including the age of the donor, have been assessed. Although the age of the donor seems to be less crucial for specific properties such as tenogenic potential [6], MSCs from aged donors generally present lagged capability in proliferation, differentiation, and immunoregulation; subsequently, aged cells showed impaired therapeutic outcomes in the disease model [7]. The infusion of aged MSC would rather deteriorate the disease severity by causing “inflammaging” in the body of recipients [8]. Senescent cells are known to display a senescence-associated secretory phenotype (SASP) that contributes to the progress of aging of neighboring cells, impaired regenerative function, and immune cell recruitment after administration [9]. One of the options to address this issue is to use MSCs derived from byproducts at delivery such as umbilical cord (UC), umbilical cord blood (UCB), and Wharton’s jelly (WJ), which possess more primitive properties than the other adult stem cells [10].

Another strongly suggested problem is the individual difference between MSCs based on the variable backgrounds from donor to donor. Moreover, MSCs from patients with specific diseases show downregulation of cell function such as an anti-inflammatory secretome, reflecting inferior therapeutic capability [11]. To overcome the limitation, disease-specific MSC selection before the application has been required. Lee et al. have demonstrated that therapeutically effective and ineffective clones have different gene expression profiles, and among the genes expressed in effective clone, endothelin-1 (EDN1) significantly increased the therapeutic results of UCB-MSCs against myocardial infarction (MI) by expressing Cadherin 2 (CDH2) and VEGF [12]. We also revealed that UCB-MSCs have donor-dependent individual differences, and hypoxic preconditioning, a promising tool for MSC targeting cardiovascular diseases, was applied to improve the therapeutic function of those cells to ischemic diseases [13]. As a result, UCB-MSCs isolated from different donors did not show the same response to hypoxic preconditioning. On the basis of genome-wide gene expression analysis, it illustrated that more effective UCB-MSC displayed distinctive expression patterns of specific genes including ANGPTL4, ADM, SLC2A3, and CDON after hypoxic preconditioning, and the expression pattern represents the pro-angiogenic property of UCB-MSCs, suggesting general indicators to guarantee successful stem cell therapy. Ragni and colleagues also recently proposed through sequential publications that validation of reference genes is a crucial step for donor selection, and then several miRNAs (miR-22-5p, miR-29a-5p, miR-26a-5p, and miR-16-5p) performed a role as reliable reference genes for selecting extracellular vesicles (EVs) from IFN-γ-pretreated adipose tissue (AT) MSCs for the treatment of osteoarthritis [14]. Accordingly, the development of disease-specific screening criteria and selection based on the criteria are still needed before the actual implantation of MSCs; even the enhancement methods would be applied. Furthermore, strategies for the improvement of the consistency and efficacy of MSCs have to be qualified whether the method is effective for the specific diseases and cells.

### Preconditioning of MSCs

MSC has plasticity; thus, many researchers and physicians in the field have tried to fine-tune the features of the cells to be suited for the targeted diseases before cell application. Cues that manipulate the features of MSCs include cytokines/chemokines, growth factors, receptor agonists, hormones, drugs, and hypoxic environment.

#### Cytokines and growth factors

Transplanted MSCs could perceive and subsequently respond to the microenvironment such as regional inflammatory signals, known as “MSC licensing.” Priming with cytokines/chemokines or growth factors released under pro-inflammatory conditions amounts to the majority of the preconditioning method (Table 1). Pretreatment with pro-inflammatory cytokines, IFN-γ, or TNF-α becomes a conventional tool to improve the therapeutic efficacy of transferred MSCs. IFN-γ priming confers the increased secretion of immunomodulatory molecules including PGE_2_, HGF, TGF-β, and MCP-1 [15]. Notably, IFN-γ-primed MSCs have a role in reclaiming immune homeostasis by inhibiting immune effector cells and promoting alternative types of immune cells. For example, IFN-γ pre-stimulation enables BM-MSCs to secrete more programmed cell death-1 ligands (PDL-1) that suppress T cell proliferation and subsequent secretion of T_H_1 cytokines [16]. MSCs preconditioned with IFN-γ reduced the frequency of T_H_17 cells and secretion of IFN-γ and TNF-α during co-culture with lymphocytes. Conversely, the result showed increased secretion of IL-6 and IL-10 and promotion of Tregs [17].

**Table 1:**
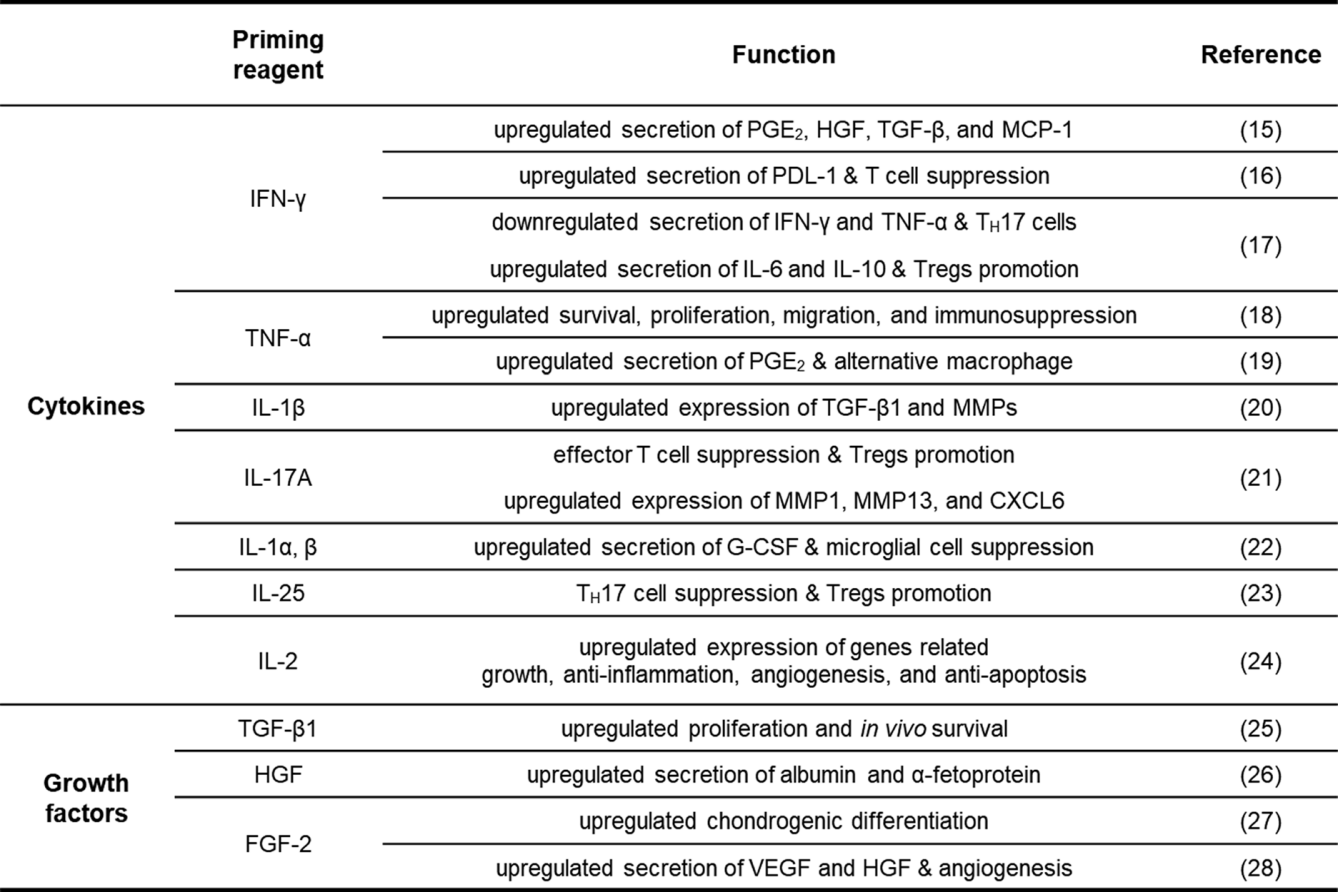
Priming effect of cytokines and growth factors on MSCs

Although it would be potentially immunogenic and the role in immunosuppression lags slightly behind IFN-γ-priming, TNF-α stimulation, as another key pro-inflammatory factor, obviously gets involved in MSC function improvement through increased secretion of immunomodulatory factors including PGE_2_, IDO, and HGF. It is reported that TNF-α exhibits therapeutic functions such as the survival, proliferation, migration, and differentiation of activated immune cells by ligation to their receptors (TNFR1 and TNFR2), and the NF-κB signaling pathway plays a crucial role in TNF-α-mediated stimulation [18]. Accordingly, TNF-α-primed BM-MSCs begin to upregulate COX-2 to synthesize PGE_2_, which increase IL-10 expression in an alternative type of macrophages and ease allergic symptoms by reducing IgE production and histamine release [19].

MSCs more efficiently attenuate target diseases after stimulation with IL-1β by adjusting in vivo immune balance and improving stem cell migration. IL-1β-priming reportedly potentiates immunomodulation and wound healing ability by upregulating the expression of TGF-β1 and matrix metalloproteinases (MMPs) [20]. IL-17 treatment regulates the differentiation of MSCs and increases proliferation in a dose-dependent manner. IL-17A-induced BM-MSCs act as superior modulators of immunological function by suppressing effector T cell proliferation and promoting Tregs. Furthermore, IL-17A-primed cells express genes associated with migration and MSC homing including MMP1, MMP13, and CXCL6 [21]. Besides these cytokines, therapeutic functions including regulation on immune cell differentiation, cytokine secretion, and anti-aging ability are influenced by the other cytokines such as IL-1α [22], IL-25 [23], and IL-2 [24].

Growth factors have been also considered as another promising priming reagent to improve the therapeutic efficacy of MSCs. TGF-β1 enhances the proliferation and in vivo survival of UC-MSCs and subsequently ameliorates the severity of LPS-induced lung injury model [25]. BM-MSCs cultured in the presence of HGF instigate to produce albumin and α-fetoprotein (AFP), and then transplanted MSCs mitigate liver injury in CCl4-induced animal model by restoring serum albumin level and suppressing transaminase activity and liver fibrosis [26]. FGF-2 has a role for modifying the property of MSCs, for instance, it expedites chondrogenic differentiation [27]. Treatment with FGF significantly improves the angiogenic capacity of dental pulp (DP) MSCs through the production of VEGF and HGF more efficiently than hypoxic preconditioning [28].

#### Immune receptor agonists

In line with preconditioning studies using cytokines and growth factors, priming with other bioactive substances such as innate immune receptor agonists could boost the therapeutic potential of MSCs as a non-selective or non-specific priming strategy (Table 2). Given the fact that toll-like receptors (TLRs) expressed in MSCs could recognize “danger” signals, TLR3 and TLR4 have been the prominent targets and employed to improve the cellular function of MSCs by ligation of their agonists, polyinosinic:polycytidylic acid (poly I:C) and lipopolysaccharide (LPS), respectively. Upon ligation on TLR3 and subsequent activation of downstream cascades, poly(I:C) exerts to modify the paracrine pattern, increase the Notch signaling pathway, and exhibit increased immunomodulatory ability such as Treg promotion and impairment of T_H_1/17 cell expansion [29]. In addition, TLR3 activation is demonstrated to be involved with PGE_2_ expression, which refers to a crucial immunosuppression factor in BM-MSCs [30]. With these distinctive capacities, TLR3-preconditioned UC-MSC showed improved therapeutic efficacy against experimental animal models for autoimmune diseases, especially on inflammatory bowel disease (IBD) [31].

**Table 2:**
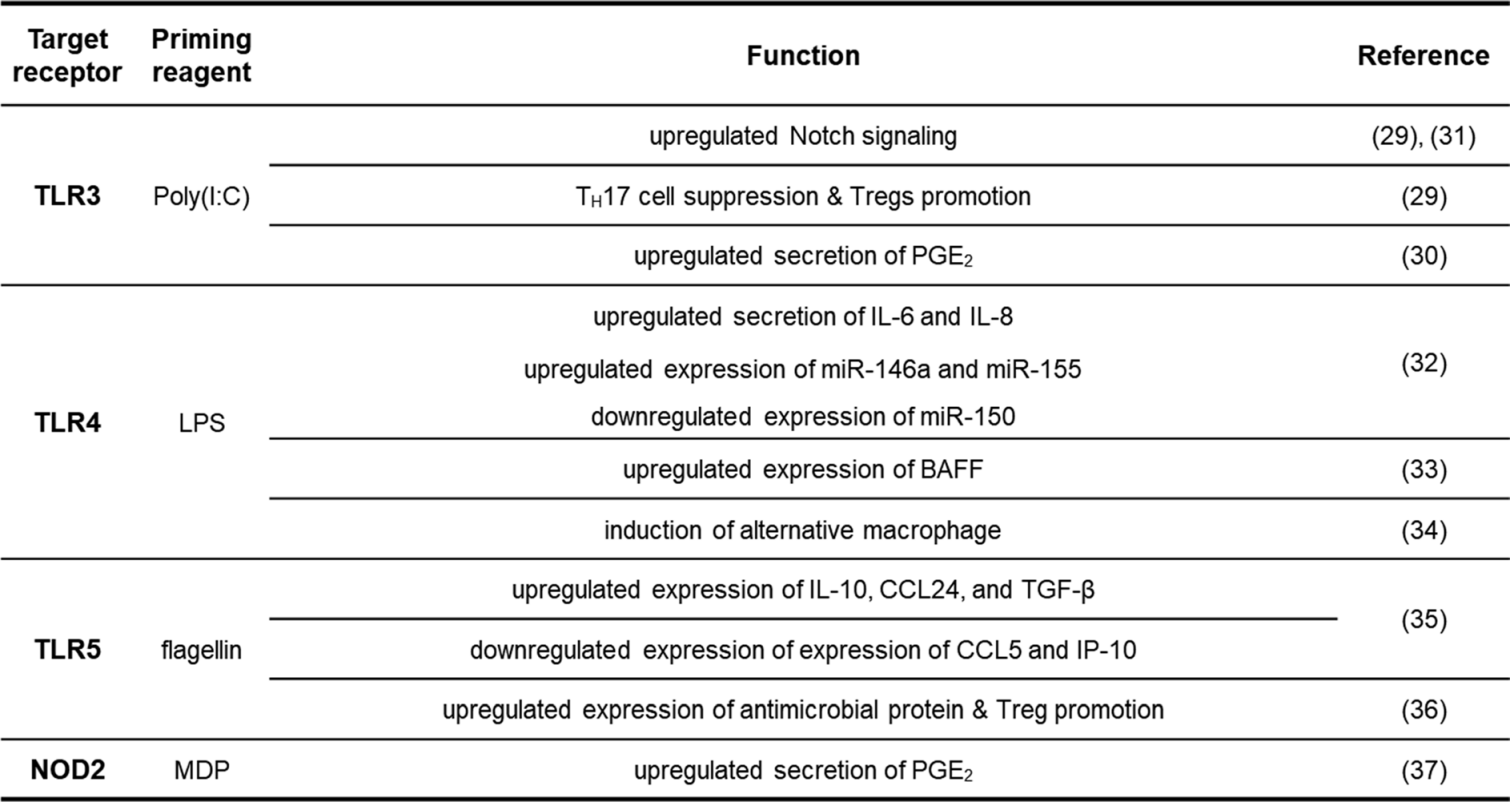
Priming effect of immune receptor agonists on MSCs

Although TLR4 activation via LPS would enforce to change MSC into a more pro-inflammatory type, the effectiveness of TLR4 priming for MSC feasibility has been still demonstrated by many researchers. Alike with other “danger” signals, functional changes mediated by LPS stimulation sustain for several days, forming short-term memory and spike when the cells encounter a second-round stimulation. Thus, transplantation of imprinted AT-MSCs had better therapeutic outcomes at promoting skin flap survival in a diabetic animal model [32]. LPS-primed BM-MSCs also have the potential for B cell-related immune regulation, since the expression of B cell-activating factor (BAFF) could be increased in response to TLR4 stimulation [33]. Also, Capitini’s group has recently suggested other uses of TLR4-primed MSCs. Exosomes from TLR4-primed UC-MSCs are known to possess a unique property to polarize monocytes/macrophages into tissue-protective type. Hence, monocytes/macrophages were cultured with exosomes from the primed cells, and these exosome-educated monocytes/macrophages (EEMs) could be applied to acute radiation syndrome instead of direct MSC injection [34].

It is demonstrated that TLR5 agonist flagellin mediates changes in the expression pattern of cytokines/chemokines by upregulating the expression of IL-10, CCL24, and TGF-β; meanwhile, the expression of CCL5 and IP-10 is reduced [35]. Flagellin preconditioning of BM-MSCs improved the therapeutic efficacy against experimental irradiation-induced proctitis by increasing the frequency of Tregs and antimicrobial protein expression while limiting apoptosis of host epithelial cells [36]. Additionally, recognition of bacterial cell wall derivative, muramyl dipeptide (MDP) by NOD2, an intracellular pattern recognition receptor promotes the immunomodulatory function of UCB-MSCs through stimulation of NOD2~COX-2 signaling and subsequently improved therapeutic effects against experimental colitis [37].

#### Hypoxia

Hypoxic preconditioning is frequently applied to strengthen the therapeutic effect of MSCs. As previous studies reported, the administration of hypoxic cultured MSCs more remarkably improves disease symptoms compared to normoxic cells. BM-MSCs increase their therapeutic potential by promoting the expression of chemokine receptors and subsequent in vivo engraftment when exposed to hypoxic conditions. Furthermore, hypoxic culture reinforces the stemness of BM-MSCs [38]. Hypoxia-inducible factor-1α (HIF-1α) has a crucial role in the upregulation of the therapeutic function of MSCs or their conditioned medium (CM) [39]. However, donor-dependent variations such as gene expression patterns must be considered even if UCB-MSCs are cultured in hypoxic conditions [13]. Up to recently, hypoxic preconditioning has been vigorously attempted to amplify the therapeutic potential of BM-MSC-derived secretory molecule including exosomes. Anderson’s group has defined that in vivo mimicking with hypoxia and serum deprivation modifies the composition of protein cargo packaged in BM-MSC-derived exosomes [40]. Indeed, Liu et al. have demonstrated that exosomes from hypoxic cultured UC-MSC could promote bone regeneration by the transfer of miR-126 [41]. Exosomes or extracellular vesicles from hypoxia-conditioned AT-MSC enhance angiogenesis through a higher level of secretome, especially VEGF [42]. In the same context, the upregulated secretory level of VEGF under hypoxic culture condition restores neuroprotective effects of aged BM-MSC against ischemic stroke [43].

#### Modification in culture method

In addition to the pretreatment of bioactive molecules and hypoxia, modification of cell culture such as 3-dimensional (3D) method holds the great possibility to improve the stemness and therapeutic potential of MSCs. Efforts to enhance yield for therapeutic cell production and cellular function have been continued by applying 3D culture priming. It is well known that contact status during cell culture causes spontaneous cell death. In the case of MSCs, cell-to-cell contact status influences its differentiation potential and immunomodulation [44]. Furthermore, the 3D culture system mimicked the original physiological property of stem cells and improved the therapeutic function as well as yield [45]. Of note, the simplest method for 3D culture is a spheroid culture. The spheroid culture of MSCs is known to enhance their therapeutic potential including anti-inflammatory properties and pro-angiogenic function [46]. 3D spheroid culture enhanced the secretion of several immunomodulatory factors, such as TGF-β1, PGE_2,_ and IL-6, and this effect could be augmented by exposure to pro-inflammatory cytokines [47]. Among the constructive supporting materials, hydrogels have drawn tremendous attention in recent years. Lee et al. have revealed that 3D culture priming with hyaluronic acid (HA)-containing hydrogels facilitates efficient and rapid retroviral gene transduction of AT-MSCs by accelerating cell cycle synchronization [48]. Moreover, 3D culturing in gelatin-based hydrogels makes MSCs improve endochondral ossification, mediating potential bone healing property [49]. The possibility has been suggested that gingival recession could be alleviated by results from a clinical study using WJ-MSCs cultured on PCL [50]. Lastly, ultraviolet B (UVB) radiation preconditioning improves the hair growth-promoting effects of AT-MSCs by generating reactive oxygen species (ROS) [51].

#### Advanced strategy for MSC preconditioning

The crosstalk between disease-specific risk factors such as a robust activation of effector immune cells and MSCs would provide crucial clues for identifying the therapeutic mechanism of MSCs and developing the disease-specific stem cell therapy. For example, activation of T_H_2 cell, B cell, and mast cell plays a pivotal role in the pathogenesis of atopic dermatitis (AD) as key effector cells in hypersensitivity and allergic reaction [52]. Among the secretory molecules, histamine is reported to activate BM-MSC, upregulating the secretion level of IL-6 [53]. Pre-exposure to these molecules is expected to boost the therapeutic function of MSC when the cells encounter the molecules again in vivo. Indeed, we elucidated that pretreatment of histamine-enriched mast cell granule stimulates UCB-MSCs to ameliorate the symptoms of experimental AD more efficiently via upregulating immunomodulation and tissue regeneration [54]. Therefore, it would be proposed priming with substances of the effector cells, instead of typical pro-inflammatory cytokine including IFN-γ and TNF-α, as an enhancement strategy for MSC-based therapy aimed at reducing allergic response and chronic inflammation in AD. This approach may be applied to other diseases by analyzing the key effector molecules in the disease pathogenesis and expected to provide customized MSCs suited to treat target diseases.

### Genetic manipulation of MSCs

Genetic modification of MSCs can be employed to improve the therapeutic potency of MSCs independently with exogenous stimuli. A number of genes related to the therapeutic function of MSCs can be a target for sustained and enhanced expression. Overexpression of VEGF in BM-MSCs promotes angiogenesis and ameliorates brain infarction [55]. With Bcl-2, VEGF overexpression improves cell survival and paracrine effect of the cells [56]. To ensure the effect of hypoxic preconditioning, HIF-1α can be transduced to BM-MSCs and emulate the therapeutic effects without any exposure process [57]. Genetic modification of BM-MSCs aiming to increase prostaglandin I synthase (PGIS) gene expression more successfully protects damaged heart and restore cardiac function in MI mouse model [58]. In addition to these, therapeutic genes including IL-4, IL-10, TGF-β1, GATA-4, and CXCR4 are utilized to increase cell survival and therapeutic effects [59].

Recently, advanced technology using clustered regularly interspaced short palindromic repeat (CRISPR)/Cas9 RNA-based nucleases facilitates more convenient and detailed genetic editing at specific desired sites. CRISPR-targeted genome editing enables MSCs to increase survival rate and alter differentiation preference [60, 61]. In addition, with this technology, MSCs could be genetically engineered to suppress the expression of certain miRNAs, known to induce osteoporosis in patients with DM [62]. Hu et al. demonstrated that CRISPR/Cas9-induced knockout of Keap1 improved anti-oxidation in AT-MSCs [63]. Introduction of CRISPR/Cas9-edited sRAGE secreting UCB-MSCs reportedly alleviated neuronal degeneration and improved homing to the lesion in Parkinson’s disease animal mice [64].

However, although stable and intensive potency can be guaranteed, genetic manipulation of MSCs is unfit to be applied to an actual application in the clinical field. Critical safety issues may be raised for the clinical use of genetically modified MSCs. Consistent activation of the specific gene would be a major cause for the development of stem cell-derived malignant tumors. Therefore, efforts for transient modification for therapeutic potential improvement are still needed. Transient epigenetic modification by chemicals has been also considered as one of the targets. Our group has made efforts to improve the MSC fundamental property and the therapeutic efficacy by modulating epigenetic mechanisms including DNMT inhibition [65]. Additionally, provisionary downregulation by using shRNA [66] or non-viral gene delivery with priming reagent [67] might be a good tool to avoid unwanted perpetual changes.

### Co-administration with supportive materials

The focus of recent studies has moved to the development of co-administrative assistant substances to increase the therapeutic function of MSCs. Co-administration with immunosuppressants or advanced materials is strongly recommendable because it does not require additional preparatory steps, such as cell priming or genetic manipulation; thus, it is convenient to apply for clinical use. Moreover, potent risks such as tumor formation and contamination of a heterogeneous population can be reduced. Bio-engineering with scaffold takes a big part in improvement methods for MSC-based therapy. Bioactive reagents such as ECM and hydrogel are used to build a structure of tissue or organ using 2D patches or 3D printed architecture. The method encourages cell-to-cell communication as shown in the spheroid culture [68]. Besides, the use of scaffolds could increase the biophysical properties of MSCs such as homing [69] and lineage determination [70]. In addition, preclinical studies using biocompatible advanced materials such as gold nanoparticle and graphene derivatives have been actively conducted [71].

### Co-treatment with promising drugs

Co-application of MSCs with immunosuppressants including rapamycin and tacrolimus showed improved therapeutic outcomes through the synergism of each remedy, by which improving the survival time in the transplantation of MSCs and reducing the adverse effects of medicines. Importantly, the application of bioactive reagents that facilitates homeostasis of in vivo immune balance stabilizes the mode of action of MSCs. For example, innate immune stimulator, MIS416 improves the immunomodulation of UCB-MSCs, recovers immune homeostasis in the gut, and, thus, boosts the therapeutic function of UCB-MSCs against experimental colitis [72]. Recently, Hu et al. have demonstrated the synergistic effect of BM-MSC and botulinum toxin type A to treat hypertrophic scars, downregulating related mRNA and protein levels including alpha-smooth muscle actin (α-SMA) [73]. Compared to both monotherapies, combined treatment with *N*-acetylcysteine (NAC) and MSC exerts better therapeutic effects on the resolution of inflammation or apoptosis in the interstitial cystitis experimental model [74].

## Future perspectives and concluding remarks

In the present study, we propose several complementary methods for improving the therapeutic efficacy of MSCs. Each method targets different preparatory steps for a cell application; hence, these findings might contribute to establishing comprehensive enhancement strategies by combinatorial use of each developed method. A combination of 2D (e.g., priming) and 3D (e.g., spheroid culture) aids complements the therapeutic effects of MSCs. For example, MSCs modified to enhance proliferation and survival are inserted into the biocompatible scaffold and the complex implanted to the damaged joint with TNF-α inhibitor to treat degenerative arthritis. In addition, biomedical technologies at the cutting edge such as gene therapy or monoclonal antibody medicines are considered for combinatorial treatment with MSCs. Indeed, Park et al. recently suggested a brand new function improvement method called in vivo priming. In their study, the authors transduced BM-MSCs to consistently secrete HGF and the engineered cells were seeded on 3D patch mixing with naïve cells resulting in the improvement of therapeutic function compared to naive MSCs [75]. Therefore, optimization of the combinatorial use of each strategy could be envisioned to maximize the therapeutic outcome of MSC therapy.

Moreover, several limitations including functional quiescence after the application and donor-dependent variation still need to be addressed in further research. To do so, we would suggest “customized clinical strategy,” which is specific for the implanted cells and disease-related environment to overcome the current obstacles to MSC-based therapy and subsequently achieve improved therapeutic outcomes. Disease-specific priming takes a big part of the “customized clinical strategy,” as we discussed above. Moreover, as a point of those tailored tactics, the time point of cell administration can be adduced. Hence, the disease-specific immune status of a patient is very important for determining the time for delivery of MSCs, since the immunomodulation ability of MSCs would be mediated by inflammatory milieu, and the responsiveness of MSC could be maximized when infused at the peak of inflammation [76].

In conclusion, despite its known limitations, MSCs still hold a considerable promise as an alternative therapeutic reagent for various rare diseases. Therefore, further researches on the disease-specific status and conditions are needed, and MSC-based therapy has to be modified suitable for each targeted disease. Moreover, the development of combination with established enhancement methods, involved from isolation to actual application, could bring the expansion of knowledge about the mechanisms of MSC-based therapy, provide novel enhancement strategies, and presumably help the patients suffering from diseases.

## Data Availability

Not applicable.

## Abbreviations

3D: Three-dimensional
AD: Atopic dermatitis
AT: Adipose tissue
bFGF: Basic fibroblast growth factor
BM: Bone marrow
CCL: CC chemokine ligand
CM: Conditioned medium
CXCL: C-X-C motif chemokine
CXCR: C-X-C motif chemokine receptor
DM: Diabetes mellitus
DP: Dental pulp
EGF: Epidermal growth factor
GM-CSF: Granulocyte-macrophage colony-stimulating factor
HGF: Hepatocyte growth factor
HIF-1α: Hypoxia-inducible factor-1alpha
IDO: Indoleamin 2,3-dioxygenase
IFN-γ: Interferon-gamma
IGF-1: Insulin-like growth factor-1
IL: Interleukin
iNOS: Inducible nitric oxide synthase
IP-10: Interferon gamma-induced protein 10
KGF: Keratinocyte growth factor
LIF: Leukemia inhibitory factor
LPS: Lipopolysaccharide
MCP-1: Monocyte chemoattractant protein-1
MDP: Muramyl dipeptide
MiR: MicroRNA
MMP: Matrix metalloproteinases
MSC: Mesenchymal stem cell
NF-κB: Nuclear factor-κB
NOD-2: Nucleotide-binding oligomerization domain-containing protein 2
PDL-1: Programmed cell death-1 ligands
PGE_2_: Prostaglandin E_2_
Poly(I:C): Polyinosinic:polycytidylic acid
TGF-β: Transforming growth factor-beta
TLR: Toll-like receptors
TNF-α: Tumor necrosis factor-alpha
Treg: Regulatory T cell
UC: Umbilical cord
UCB: Umbilical cord blood
VEGF: Vascular endothelial growth factor
WJ: Wharton’s jelly
